# Different Forms of Ursolic Acid and Their Effect on Liver Regeneration

**DOI:** 10.1155/2020/4074068

**Published:** 2020-07-26

**Authors:** Lenka Žaloudková, Alena Tichá, Jana Nekvindová, Ladislava Pavlíková, Zdeněk Zadák, Pavel Živný

**Affiliations:** ^1^Institute of Clinical Biochemistry and Diagnostics, University Hospital Hradec Kralove, Sokolska Str. 581, Hradec Kralove 500 05, Czech Republic; ^2^Department of Research and Development, University Hospital Hradec Kralove, Sokolska Str. 581, Hradec Kralove 500 05, Czech Republic; ^3^2nd Department of Internal Medicine–Gastroenterology, Charles University, Faculty of Medicine in Hradec Kralove, Hradec Kralove 500 03, Czech Republic

## Abstract

The aim of this study was to determine the effect of natural and encapsulated sources of ursolic acid on liver regeneration. Four ursolate sources were tested. Two forms of ursolic acid encapsulates were combined with cyclodextrins, i.e., gamma-CD (gCD) and beta-CD, and two natural sources were adjusted by homogenization (HAP) and micronization of apple peel using Jonagold apples. All ursolate forms were applied intragastrically in daily doses of 20 mg for 7 days. Laboratory rats were fed with standard laboratory diet. Further, gCD and MAP were also tested with a high-fat diet (6 weeks). Partial hepatectomy (PH) was performed 24 hours before the end of the experiment. The concentration of plasma hepatocyte growth factor (HGF) was determined with an immunoassay; simultaneously, the expression of HGF and CYP7A1 in the liver was quantified through qPCR. HGF expression and plasma levels were significantly increased 24 hours after PH in both the HAP (*p*=0.038) and HFgCD groups (*p*=0.036), respectively. The correlation between HGF expression and plasma values was significant (*p*=0.04). The positive effects on liver regeneration were found in both the gCD and HAP forms of ursolic acid, whose effects were confirmed through the upregulation of HGF.

## 1. Introduction

Ursolic acid is classified within the group of triterpenes which, along with steroids, belongs to the group of isoprenoids whose biosynthesis is based on squalene. To date, >1,000 triterpenoids with an incredible range of biological activities have been isolated from natural sources. Among these, ursolic acid has biological properties such as an anti-inflammatory, anticancer, antiangiogenic, and antioxidative agent [1, 2].

Ursolic acid (UA) is a pentacyclic triterpenoid found in several herbs, spices, and fruits like rosemary, thyme, apple, and olive. [3]. The consumption of apples has been linked to the prevention of various chronic disease, and apple peel exhibits more potent antioxidant and antiproliferative activities than apple flesh. Apple peel is a suitable source of UA, and these fruits are well available locally. Triterpenes content in various fruits and plants was confirmed the highest UA contain with lavender, rosemary, and apples, especially in the Jonagold variety [3]. The bioavailability of ursolic acid is problematic due to its hydrophobic characteristics. Its encapsulation with cyclodextrins or its increased bioavaibility through micronization has facilitated the intake of ursolic acid. In this regard, most researchers use organic solvents (e.g., acetone, ethanol, and dichloromethane) to dissolve ursolic acid or used organic solvents during preparation, which may be hazardous to humans. Further, its bioavailability has been assessed in the form of ursolic acid stearoyl glucoside [4], different phospholipid complexes [5], or nanoparticles [6]. When UA is combined with cyclodextrins, its solubility in water is greatly enhanced by the cyclic structure of the latter [7]. Inspite of possible bioavailability improvement, we have attempted to increase water solubility of ursolic acid by encapsulation. Cyclodextrins are often used in the pharmacology and food industry as additive compounds.

Partial hepatectomy (PH) has been shown as the best method to test liver regeneration [8–10]. After the procedure, the remaining liver tissue is exposed to increased blood flow, and cell replication begins after 24 hours. Immediately after resection, the expression of urokinase plasminogen activator (uPA) is upregulated throughout the entire liver tissue, starting as early 5 minutes after PH, activating the expression of matrix remodelling proteins such as the hepatocyte growth factor (HGF) [11]. The latter is a direct mitogen capable of inducing liver proliferation in an independent manner; however, there are other proteins classified as auxiliary liver mitogens (e.g., TNF alpha, IL-6) [8, 9]. HGF is crucial for hepatic regeneration [8, 10]; on the other hand, the effect of IL-6 in hepatic regeneration has not been confirmed so far [12].

The aim of this study was to verify the effect that both natural and encapsulated sources of ursolic acid have on liver regeneration as well as to confirm a reliable biochemical blood marker indicating liver regeneration.

## 2. Study Design

### 2.1. Animals

Male Wistar rats (220–320 g in weight; 7 weeks of age) were purchased from BioTest (Konarovice, Czech Republic). The animals were housed at 22 ± 2°C, 55 ± 10% humidity, and 12 hours light/dark cycles. The animals had access to water and standard laboratory pellet diet ad libitum (Velaz, Czech Republic). The study protocols were approved by the Ethical Expert Committee of the Medical Faculty, Charles University Hradec Kralove (protocol No. MSMT-42093/2015-5). All experiments were in adherence to rule No. 246/1992 for the protection of animals against cruelty. The rats were divided into 7 groups (*n* = 6 per group) after 1 week of acclimatization.

### 2.2. Animal Groups

  CTRL: standard pellet diet for 7 days–Control group
  bCD: standard pellet diet for 7 days and beta-cyclodextrine ursolic acid
  gCD: standard pellet diet for 7 days and gamma-cyclodextrine ursolic acid
  HAP: standard pellet diet for 7 days and homogenate apple peel
  MAP: standard pellet diet for 7 days and micronized apple
  HFgCD: HF diet for 7 days and gamma-cyclodextrine ursolic acid
  HF-MAP: HF diet for 7 days and micronized apple peel

### 2.3. Tested Compounds

Four ursolate substances were prepared from apple peel using Jonagold variety apples. Two substances were encapsulated with cyclodextrins (CD), gamma-CD (gCD), and beta-CD (bCD). The cyclodextrin (CD) encapsulates of ursolic acid were supplied by Betulinies (Stribrna Skalice, Czech Republic). The other two substances were adjusted by homogenization (HAP; Ø > 5 *µ*m) and micronization of apple peel (MAP; Ø = 5 *µ*m) [5] and were prepared by K2pharm (Opava, Czech Republic) without using organic solvents. All forms were supplemented on a daily basis as water suspensions directly into the stomach using a gastric tube (20 mg of UA per animal). The amount of ursolic acid was determined and verified after extraction of apple peel into ethylacetate by using GC-MS [13], and the amount of ursolic acid was 5.6 g in 100 g of Jonagold variety apple peel.

### 2.4. Diet

The animals were fed with a standard diet for 1 week before the study and then divided into 7 groups at random (*n* = 6 per group). The standard diet contained 21.1% protein, 5.1% fat, 60.0% carbohydrates, 3.9% fiber, 7.9% minerals, and 2.0% vitamins.

A high-fat diet (HFD) was prepared with 35.5% fat (beef tallow), 59% protein and carbohydrates, and 5.5% minerals and vitamins (Weber, Czech Republic). The corresponding animal groups were fed with this high-fat diet for 6 weeks before experiment.

### 2.5. Experimental Design

Partial hepatectomy (PH) (70%) was practiced 24 hours before the end of the experiment according to Higgins and Anderson [14]. The animals were anesthetized using ether inhalation before surgery and placed in cages upon awakening. The last doses of ursolic acid were supplemented after PH. The animals were later overdosed with an ether anaesthetic 24 hours after PH and sacrificed by exsanguination from the abdominal aorta. The liver lobes were excised during partial hepatectomy and, the liver remnants were weighed, frozen, and stored for posterior analysis.

## 3. Methods

Blood samples were collected into a heparin-coated tube from the retro-orbital sinus before the administration of ursolic acid and before PH (Sarstedt, Germany). Blood samples were also collected at the end of the experiment. The blood cells were precipitated by centrifugation, and aliquots of plasma were stored at −20°C for posterior analysis. The concentration of aspartate aminotransferase (AST), alanine aminotransferase (ALT), total cholesterol (tCH), and triglyceride (TAG) was measured using a Cobas 8000 instrument (Roche, Basel). The concentration of hepatocyte growth factor (HGF) in plasma was determined with the sandwich enzyme immunoassay cloud kit (Clone Corporation, USA). Total bile acid (BA) was determined with an enzymatic cycling method (Diazyme Laboratories, USA). Liver weight was calculated by the following equation [15]:

(1) $$\begin{array}{c} \text{hepatic r}e\text{generation rate}\left(\text{HRR}\right)\left(\%\right)=\frac{C-\left(A-B\right)}{A\times 100}, \end{array}$$

where *A* is the estimated total liver weight at the time of the partial hepatectomy, *B* is the excised liver weight, and *C* is the weight of the regenerated liver at sacrifice.

### 3.1. RNA Isolation and qPCR

Total RNA was extracted from ∼30 mg of snap frozen liver tissue samples using TRIzol (Invitrogen, USA). RNA concentration was determined with a Nanodrop ND1000 spectrophotometer (Thermo Scientific, USA) at 260/280 nm. RNA integrity was determined with an Agilent bioanalyzer 2100 and RNA 6000 Nano kit (Agilent Technologies, USA). cDNA was synthesized using the Transcriptor First-Strand cDNA Synthesis kit (Roche) using 1 *µ*g of total RNA and a 1 : 2 ratio of dT and random hexamer primers according to the manufacturer's protocol. Subsequently, 25 ng cDNA was included in the qPCR reaction using the 1X Gene Expression Master Mix and 1X TaqMan-labeled primers (Table 1); the PCR reaction was performed in a RotorGene *Q* real-time PCR instrument (Qiagen, Netherlands). The housekeeping gene HPRT1 was used as an expression reference. The standardized data were used to calculate relative gene expressions using the 2^−ΔΔCT^ method [16].

### 3.2. Statistical Analysis

Analyses were performed with the SigmaStat Software (Systat, USA). First, normality was tested by the Kolmogorov–Smirnov test. In normal data, comparisons were made among the groups using one-way ANOVA followed by Tukey–Kramer's post hoc test. The results are expressed as the mean ± SD. In the case of non-Gaussian distribution, nonparametric Kruskal–Wallis test and Dunn's post hoc test were used. Data are expressed as median values (interquartile range). Significance was set at *p* < 0.05.

## 4. Results

The plasma markers of lipid metabolism and liver enzyme activity were evaluated before administration of UA and after the administration of UA and after PH (Table 2). The concentrations of plasma TAG and tCH were significantly reduced following the administration of ursolate compounds in the HF diet group (Table 2). Further, the catalytic activity of ALT and AST confirmed that all UA forms result in the same level of activity in comparison with the CTRL and did not induce liver damage.

**Figure 1:**
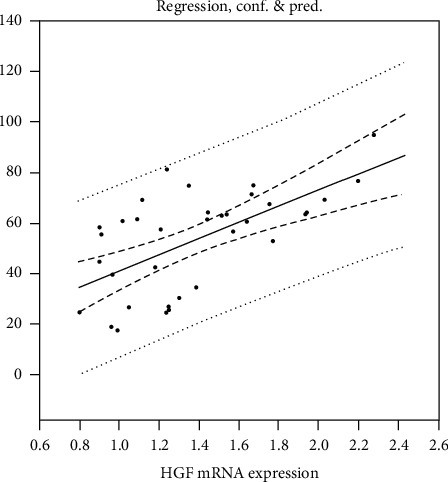
Spearman correlation of HGF expression in the liver and plasma levels.

The level of tCH after PH (Table 2) was significantly higher in the HF diet groups. The remaining groups did not show hepatic impairment and the lipid metabolism was not different either except for the HF-MAP group where ALT activity was significantly increased. Moreover, significantly increased concentrations of tCH and TAG were observed in the plasma of the HF diet groups after PH in comparison with the CTRL. HGF expression in the liver and plasma levels is shown in Table 3. CYP7A1 expression in the liver and plasma levels of BA are shown in Table 4. The Spearman correlation is shown in Table 5 and Figure 1.

HGF expression and plasma levels were significantly increased 24 hours after PH in the HAP and HFgCD groups (Table 3). The correlation between HGF expression in liver and plasma levels was confirmed by Spearman's correlation (*p* < 0.05) (Table 5, Figure 1). HGF expression and plasma levels are in agreement with HRR. Further, the levels of BA were lower in the MAP and HF-MAP groups (Table 4); however, it did not block the BA flow.

## 5. Discussion

In the present study, we evaluated the effect of UA on liver regeneration after its administration in various forms and without the use of an organic solvent adjuvant. The effects of UA cyclodextrins and UA micronization were evaluated under normal and high-fat diets, finding significantly increased levels of in TAG and tCH in the plasma of the evaluated rats in the latter condition (Table 2). Our data clearly showed (Table 2) that, after 6 weeks, HFD TAG and tCH were increased in plasma; however, there was a significant decrement in these levels after 7 days of UA administration. These results are consistent with the known hypolipidemic effects of UA [17, 18] in mice. In addition, no liver damage was caused by the tested UA sources as shown by the enzyme activity levels of ALT and AST after the administration of UA and after partial hepatectomy.

Significantly lower levels of TAG in rats after the administration of ursolic acid could be caused by an increased lipoprotein lipase activity; despite an HF diet, these decreased TAG and tCH levels could be observed during the 7 days of gCD and MAP administration (Table 2), which was confirmed in all of the experimental groups. Further, liver tCH was increased after PH due to the changes in energy available for cell proliferation. The HFgCD and HF-MAP groups showed a significantly higher concentration of tCH and TAG in plasma such as HGF expression, supporting the hypothesis that early lipid accumulation in the liver after PH could be beneficial for its regeneration [19, 20].

DNA replication was markedly reduced after PH while BA flow was blocked. Plasma HGF values were also decreased in the absence of BA; on the other hand, HGF concentration was increased when BA flow was restored [8, 9, 12]. The levels of bile acids (BA) in our study groups were similar with significant differences only a diet with MAP or HF-MAP (Table 4). This indicates minimal BA liver load after PH, showing no blockage of BA outflow in either study group.

The ursolic acid preparations confirmed increased liver regeneration in comparison to the CTRL group. HGF expression in liver tissue and plasma levels were significantly increased 24 hours after PH in the HAP and HFgCD groups compared with those of the CTRL group, with both values being significant. HGF expression in the liver confirmed a significant increment in HF-MAP. The results (Table 3) are consistent with the calculated liver regeneration [15], and increased HRR in the HF diet may also be related to fat accumulation [20] in the liver. When comparing groups with the SLD diet, the results of both plasma HGF and hepatic expression HGF and HHR are consistent with those of [21], which administered ursolic acid as a chemical substance, observing a significant liver to body weight increment along with the upregulation of cyclins and C/EBP proteins [21].

The correlation results of plasma HGF and HGF expression are shown in Table 5 and Figure 1. We suggest that plasma HGF is a suitable marker indicating the rate of liver regeneration, which can be easily monitored through an ELISA. The biological activity of the tested forms of UA showed HAP as the most suitable source according to HGF expression, although gCD also showed acceptable results. It can be assumed that the homogenized peel of Jonagold apples is the most suitable source of ursolic acid without the need of toxic chemical solvents. Taken together, our findings are consistent [8, 9, 21] and show HGF as a mitogenic substance with major effects on liver growth. We have shown that ursolic acid stimulates DNA synthesis, and at the same time, it displays hepatoprotection against apoptosis induce by high HGF levels.

## 6. Conclusion

The effects of ursolic acid extracted, micronized, and homogenized from apple peel on liver regeneration has not been previously described elsewhere. Interestingly, this ursolic acid extract had the same effect on liver regeneration observed with encapsulated gamma cyclodextrins. Therefore, suitable sources of ursolic acid for liver regeneration can now be extracted without the need of organic solvents. In addition, this study revealed that plasma HGF is a suitable biochemical marker of liver regeneration.

## Figures and Tables

**Table 1:** Primers.

Assay ID	Gene	Gene name
Rn00566673_m1	Hgf	Hepatocyte growth factor
Rn00564065_m1	Cyp7a1	Cytochrome P450, family 7, subfamily a, polypeptide 1

**Table 2:** The plasma markers of lipid metabolism and enzyme activity during the study.

	Groups							
Analytes	Time	CTRL	bCD	gCD	HAP	MAP	HFgCD^*∗*^	HF-MAP^*∗*^
ALT (*µ*kat/l)	T1	0.9 (0.81–0.98)	1.02 (0.86–1.08)	0.8 (0.69–0.87)	0.78 (0.74–0.86)	0.78 (0.69–0.95)	0.81 (0.73–0.94)	0.91 (0.85–0.98)
	T2	0.89 (0.78–0.99)	1.01 (0.86–1.16)	0.75 (0.62–0.89)	0.81 (0.73–0.91)	0.78 (0.69–0.93)	0.76 (0.702–0.90)	0.94 (0.83–1.05)
	T3	7.03 (5.62–8.15)	4.51 (3.72–5.86)	7.34 (5.60–7.98)	5.12 (3.56–5.92)	4.97 (3.44–5.28)	5.05 (3.59–7.32)	10.54 (9.85–11.24)^a^

AST (*µ*kat/l)	T1	1.43 (1.36–1.89)	1.55 (1.50–1.95)	1.37 (1.20–1.74)	1.46 (1.38–1.82)	1.31 (1.25–1.60)	1.48 (1.24–1.79)	1.36 (1.23–1.44)
	T2	1.66 (1.30–1.87)	1.61 (1.31–1.89)	1.45 (1.20–1.73)	1.44 (1.31–1.68)	1.38 (1.24–1.62)	1.50 (1.28–1.78)	1.39 (1.25–1.50)
	T3	7.91 (6.92–10.02)	6.36 (5.45–8.83)	7.85 (4.93–10.62)	9.16 (5.97–11.01)	8.43 (7.12–9.85)	6.75 (5.74–9.38)	12.29 (10.21–13.77)

tCH (mmol/l)	T1	1.46 (1.31–1.58)	1.32 (1.24–1.36)	1.49 (1.34–1.56)	1.42 (1.28–1.58)	1.37 (1.30–1.8)	2.21 (2.01–2.30)^a^	2.24 (2.10–2.29)^a^
	T2	1.43 (1.25–1.62)	1.34 (1.21–1.39)	1.45 (1.38–1.56)	1.47 (1.37–1.72)	1.54 (1.37–1.72)	1.73 (1.54–1.88)^b^	1.42 (1.31–1.49)^b^
	T3	0.99 (0.94–1.02)	0.99 (0.93–1.02)	1.01 (0.82–1.14)	0.95 (0.65–1.00)	0.92 (0.90–1.06)	1.86 (1.74–2.00)^a,c^	1.04 (1.01–1.16)

TAG (mmol/l)	T1	2.19 (1.86–2.31)	1.59 (1.52–2.02)	1.78 (1.67–2.04)	1.76 (1.62–2.03)	1.71 (1.65–1.88)	3.81 (2.86–4.77)^a^	3.73 (3.01–4.56)^a^
	T2	2.03 (1.92–2.03)	1.56 (1.42–1.73)^a^	1.79 (1.58–1.90)^a^	1.73 (1.61–1.91)^a^	1.11 (0.90–1.38)^a^	2.09 (2.01–2.93)^b^	1.92 (1.64–2.51)^b^
	T3	0.46 (0.43–0.62)	0.46 (0.42–0.53)	0.49 (0.38–0.68)	0.36 (0.31–0.54)	0.56 (0.44–0.62)	0.79 (0.67–0.91)^c^	0.64 (0.57–0.72)^c^

BA (*µ*mol/l)	T1	33.53 (22.01–36.22)	18.85 (15.31–28.32)	23.89 (16.73–32.70)	18.29 (15.38–26.38)	15.70 (11.55–23.82)	15.80 (10.67–23.67)	18.56 (15.73–32.51)
	T2	30.19 (23.81–36.95)	17.86 (15.09–29.81)	23.07 (19.21–30.65)	19.85 (15.76–28.49)	16.43 (11.92–27.96)	14.18 (10.96–24.78)	19.71 (13.08–25.50)
	T3	97.04 (85.72–124.73)	111.49 (108.01–156.29)	123.52 (94.19–156.94)	74.05 (57.54–116.35)	40.88 (35.17–42.10)^a^	92.78 (74.51–112.01)	39.16 (32.71–45.61)^a^

T1: biochemical markers of lipid metabolism before administration of ursolate compounds. T2: biochemical markers of lipid metabolism after administration of ursolate compounds. T3: biochemical markers of lipid metabolism 24 hours after PH. ^a^*p* < 0.05 vs CTRL, ^b^*p* < 0.05 vs T1 (before administration of ursolate compound), ^c^*p* < 0.05 vs CTRL, bCD, gCD, HAP, and MAP). CTRL: control group, bCD: beta-cyclodextrine ursolic acid, gCD: gamma-cyclodextrine ursolic acid, HAP: homogenate apple peel, MAP: micronized apple peel, HFgCD: HF diet and gamma-cyclodextrine ursolic acid (HFgCD), HF-MAP: HF diet and micronized apple peel, AST: aspartate aminotransferase, ALT: alanine aminotransferase, tCH: total cholesterol, TAG: triglyceride, BA: bile acid, ^*∗*^HF diet: high-fat diet.

**Table 3:** HGF expression in the liver and concentration in plasma after PH and HRR.

Group	CTRL	bCD	gCD	HAP	MAP	HFgCD	HF-MAP
HGF (ng/l) in blood	31.46 (25.05–42.82)	43.58 (36.22–48.06)	55.76 (41.91–59.83)	62.67 (57.11–68.52)^a^	43.56 (34.36–49.62)	63.58 (56.88–78.83)^a^	41.96 (34.04–52.87)
HGF expression [16]	1.108 (1.09–1.203)	1.097 (1.02–1.193)	1.260 (1.16–1.315)	1.975 (1.815–2.109)^a^	1.037 (0.987–1.126)	1.341 (1.256–1.497)^a^	2.178 (1.893–2.226)^a^
HRR (%) [15]	11.4 (8.5–14.1)	15.4 (12.5–17.6)	14.5 (12.8–17.1)	17.4 (15.8–19.9)^a^	15.5 (12.7–17.1)	17.7 (16.1–19.7)^a^	17.3 (15.9–18.9)^a^

^a^ *p* < 0.05 vs CTRL, HGF: hepatocyte growth factor, hepatic regeneration rate (HRR) (%): [*C*−(*A*−*B*)]/A × 100. *A*: estimated total liver weight at the time of the partial hepatectomy, *B*: the excised liver weight, and *C*: the weight of the regenerated liver at sacrifice.

**Table 4:** CYP7A1 expression in the liver and bile acid concentration in plasma after PH.

Group	CTRL	bCD	gCD	HAP	MAP	HFgCD	HF-MAP
BA after PH (*µ*mol/l)	97.04 (85.72–124.73)	111.49 (108.01–156.29)	123.52 (94.19–156.94)	74.05 (57.54–116.35)	40.88 (35.17–42.10)^a^	92.78 (74.51–112.01)	39.16 (32.71–45.61)^a^
CYP7A1 expression [16]	0.535 (0.397–0.672)	0.751 (0.566–0.838)	0.543 (0.409–0.596)	0.852 (0.767–0.912)	0.769 (0.627–0.803)	0.885 (0.639–0.916)	0.370 (0.315–0.458)

^a^ *p* < 0.05 vs CTRL, CYP7A1: cytochrome P450, family 7, subfamily a, polypeptide 1.

**Table 5:** Spearman correlation of HGF expression in the liver and plasma levels.

Group	R	*p* < 0.05
HGF ELISA vs. HGF expression	0.379	0.04
CYP7A1 vs. BA	−0.0484	0.78

HGF: hepatocyte growth factor, CYP7A1: cytochrome P450, family 7, subfamily a, polypeptide 1, *R:* correlation coefficient.

## Data Availability

The data used to support the findings of this study are presented within the article.

## Abbreviations

ALT: Alanine aminotransferase
AST: Aspartate aminotransferase
BA: Bile acid
bCD: Beta-cyclodextrin ursolic acid
CD: Cyclodextrines
C/EBP: CCAAT/enhancer binding proteins
CTRL: Control group
CY7A1: Cytochrome P450, family 7, subfamily a, polypeptide 1
gCD: Gamma-cyclodextrin ursolic acid
HAP: Homogenate apple peel
HF: High-fat diet
HFgCD: HF diet and gamma-cyclodextrin ursolic acid
HF-MAP: HF diet and micronized apple peel
HGF: Hepatocyte growth factor,
HRR: Hepatic regeneration rate
MAP: Micronized apple
PCR: Polymerase chain reaction
PH: Partial hepatectomy
R: Correlation coefficient
TAG: Triglyceride
tCH: Total cholesterol
UA: Ursolic acid.
